# Increased Apoptosis in the Paraventricular Nucleus Mediated by AT1R/Ras/ERK1/2 Signaling Results in Sympathetic Hyperactivity and Renovascular Hypertension in Rats after Kidney Injury

**DOI:** 10.3389/fphys.2017.00041

**Published:** 2017-02-02

**Authors:** Hongguo Zhu, Lishan Tan, Yumin Li, Jiawen Li, Minzi Qiu, Lanying Li, Mengbi Zhang, Min Liang, Aiqing Li

**Affiliations:** Department of Nephrology, Nanfang Hospital, Southern Medical University, State Key Laboratory of Organ Failure Research, National Clinical Research Center of Kidney DiseaseGuangzhou, China

**Keywords:** paraventricular nucleus, apotosis, AT1R/Ras/ERK1/2, sympathetic activity, renal hypertension

## Abstract

**Background:** The central nervous system plays a vital role in the development of hypertension, but the molecular regulatory mechanisms are not fully understood. This study aimed to explore signaling in the paraventricular nucleus (PVN) which might contribute to renal hypertension.

**Methods:** Renal hypertension model was established by five-sixth nephrectomy operation (5/6Nx) in male Sprague Dawley rats. Ten weeks afterwards, they were random assigned to no treatment, or intracerebroventricular injection (ICV) with artificial cerebrospinal fluid, losartan [angiotensin II receptor type 1 (AT1R) antagonist], farnesylthiosalicylic acid (Ras inhibitor), PD98059 (MEK inhibitor), or SB203580 (p38 inhibitor) and Z-DEVD-FMK (caspase-3 inhibitor). Before and after treatment, physiological and biochemical indices were measured. Immunohistochemistry, western blot and RT-PCR were applied to quantify key components of renin-angiotensin system, apoptosis-related proteins, Ras-GTP, and MAPKs in the PVN samples. TUNEL assay was used to measure the situ apoptosis in PVN.

**Results:** The 5/6Nx rats showed significantly elevated systolic blood pressure, urinary protein excretion, serum creatinine, and plasma norepinephrine (*p* < 0.05) compared to sham rats. The expression of angiotensinogen, Ang II, AT1R, p-ERK1/2, or apoptosis-promoting protein Bax were 1.08-, 2.10-, 0.74-, 0.82-, 0.83-fold higher in the PVN of 5/6Nx rats, than that of sham rats, as indicated by immunohistochemistry. Western blot confirmed the increased levels of AT1R, p-ERK1/2 and Bax; meanwhile, Ras-GTP and p-p38 were also found higher in the PVN of 5/6Nx rats, as well as the apoptosis marker cleaved caspase-3 and TUNEL staining. In 5/6Nx rats, ICV infusion of AT1R antagonist, Ras inhibitor, MEK inhibitor or caspase-3 inhibitor could lower systolic blood pressure (20.8-, 20.8-, 18.9-, 14.3%-fold) together with plasma norepinephrine (53.9-, 57.8-,63.3-, 52.3%-fold). Western blot revealed that blocking the signaling of AT1R, Ras, or MEK/ERK1/2 would significantly reduce PVN apoptosis as indicated by changes of apoptosis-related proteins (*p* < 0.05). AT1R inhibition would cause reduction in Ras-GTP and p-ERK1/2, but not vice versa; such intervention with corresponding inhibitors also suggested the unidirectional regulation of Ras to ERK1/2.

**Conclusion:** These findings demonstrated that the activation of renin-angiotensin system in PVN could induce apoptosis through Ras/ERK1/2 pathway, which then led to increased sympathetic nerve activity and renal hypertension in 5/6Nx rats.

## Introduction

Increased incidence of chronic kidney disease has become a major global public health problem (Schoolwerth et al., 2006). Its primary risk factors are old age, diabetes, hypertension, hyperlipidemia, and so forth, among which hypertension is also the most common secondary disease (Bash et al., 2010; Gansevoort et al., 2013). Hypervolemia and renin-angiotensin system (RAS) activation are considered major determinants of hypertension in chronic renal failure, and blood pressure can be reduced by controlling these two factors (Nguy et al., 2013).

The main hormone generated by the RAS system is angiotensin (Ang) II, which is produced by angiotensinogen (AGT) cleavage (Griffin and Bidani, 2006). Ang II binding to angiotensin II type 1 receptor robustly activates mitogen-activated protein kinase (MAPK) intracellular signaling pathways to adjust hypertension (Wei et al., 2008a). Three major terminal effector kinases of the MAPK family include extracellular signal regulated protein kinases 1 and 2 (ERK 1/2), stress-activated protein kinase/c-Jun NH2-terminal kinases, and p38 MAPK (Johnson and Lapadat, 2002). MAPK signaling pathways regulate a variety of cellular biological processes such as gene expression, and cell proliferation, differentiation, survival, and death, in normal developmental processes of the brain (Mielke and Herdegen, 2000; Chang and Karin, 2001). Overactivation of brain angiotensin-converting enzyme (ACE)–angiotensin II (Ang II)–angiotensin II type 1 receptor (AT1R) axis has been found to play a important role in the development and maintenance of hypertension and then elevated oxidative stress in brain and increased the activity of sympathetic nervous system (Veerasingham and Raizada, 2003). Moreover, an increased oxidative stress leads to the inward flow of Ca^2+^ in neurons, thus activating proapoptotic mechanism and leading to neuronal losses (Brown and Davis, 2002).

It is generally admitted that hypertension primarily results from chronic hyperactivity of the sympathetic nervous system (Gonzalez et al., 2002). There are several evidences suggest that elevated sympathetic nerve activity results in dysregulated blood pressure, causing chronic hypertension (Guyenet, 2006; Malpas, 2010). The central nervous system also plays a vital role in the blood pressure regulation in hypertension. Cardiovascular regulatory brain nuclei include the subfornical organ, PVN of the hypothalamus, rostral ventrolateral medulla (RVLM), and nucleus tractussolitarii of the brainstem (Ho et al., 2008; Braga et al., 2011).

Previous work had suggested that in the rostral ventrolateral medulla of stroke-prone spontaneously hypertensive rats, AT1R could activate caspase-3 through the Ras/p38/ERK pathway, which might be involved in the mechanisms underlying sympathoexcitation and pathogenesis of hypertension (Kishi et al., 2010). As RVLM, PVN also plays a pivotal role in regulating blood pressure in hypertensive states (Xue et al., 2012; Su Q. et al., 2014). Data from our lab also demonstrate there is RAS activation in PVN of chronic renal failure rats (Cao et al., 2015). It has been found that oxidative stress is a key mechanism in Ang-II-dependent hypertension, and Ang-II-induced ROS participate along the SFO-PVN-RVLM pathway in the regulation of hypertension (Shafton et al., 1998). Of note, some parvocellular subgroups in PVN projected to the pressor region of the RVLM and then influenced sympathetic nerve activity. Furthermore, Ang-II microinjected into the PVN directly caused cardiac sympathetic activation and higher mean arterial pressure in 2-kidney-1-clip rats than in sham rats (Han et al., 2011). Thus, we speculated the regulatory signaling that was revealed in the RVLM may also be activated in the PVN during pathogenesis of hypertension (Kishi et al., 2010).

**Figure 1:**
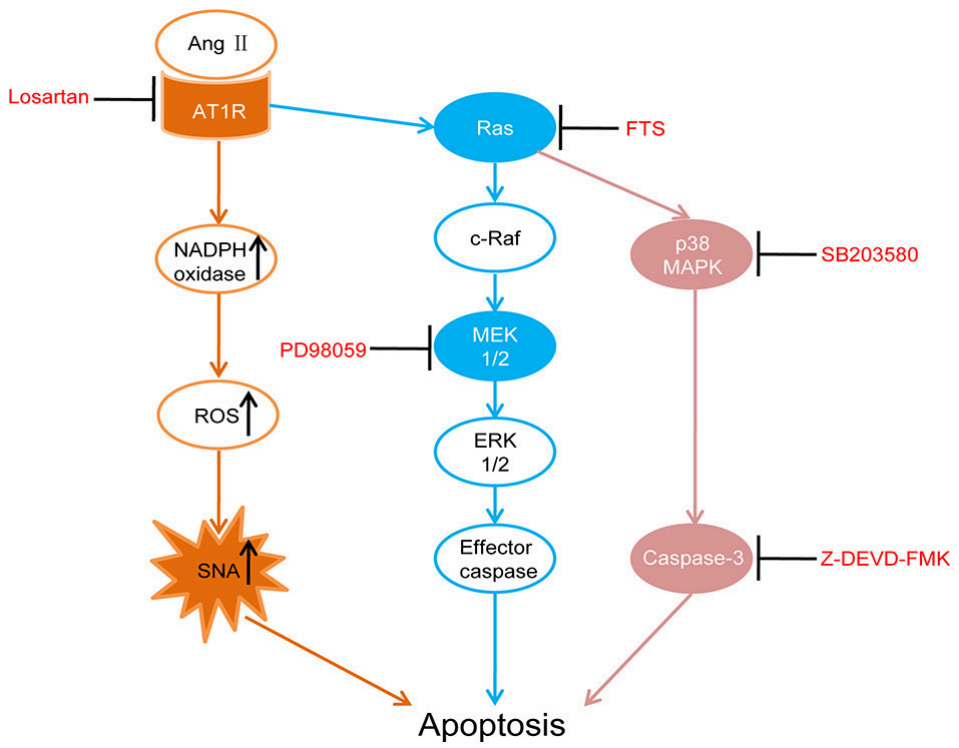
**Schematic diagram illustrates the apoptotic pathway being examined and where each drug acts**. Losartan (angiotensin II receptor type 1 (AT1R) antagonist), FTS (farnesyl thiosalicylic acid, Ras inhibitor), PD98059 (MEK inhibitor), SB203580 (p38 inhibitor), Z-DEVD-FMK (caspase-3 inhibitor).

In this study, five-sixth nephrectomy was used to establish a renal hypertension model (Badyal et al., 2003; Yamaguchi et al., 2014). We first assessed whether RAS were activated in the PVN of rat models by detecting the key components angiotensinogen, Ang II and AT1R. Then we performed intracerebroventricular (ICV) injections with AT1R antagonist (losartan), as well as Ras (farnesylthiosalicylic acid, FTS) (Gana-Weisz et al., 1997), MEK (PD98059, which effectively inhibits ERK1/2 phosphorylation) (Virdee and Tolkovsky, 1996), p38 MAPK (SB203580) (Piao et al., 2003) and caspase-3 inhibitors (Z-DEVD-FMK) (Knoblach et al., 2004) respectively in the renal hypertension rats. Those interventions were designed to examine whether there were similar associations of AT1R, Ras, and MAPKs in apoptosis regulation in the PVN as previously reported in the RVLM, which eventually led to overactive SNA and renal hypertension. In Figure 1, we illustrated the apoptotic pathway involved this research and where the various inhibitors acted (Xia et al., 1995; Downward, 1998; Braga et al., 2011).

## Materials and methods

### Animals and grouping

All animal experiments were approved by the Institutional Animal Care and Use Committee of Nanfang Hospital, Southern Medical University, and carried out in accordance with the ARRIVE guidelines (McGrath et al., 2010). The study protocol was approved by the Ethics Committee for experimental animals, Southern Medical University.

Male Sprague-Dawley rats (180–200 g) were maintained under a 12/12 h light/dark cycle at 22°C, and fed a standard laboratory chow *ad libitum*. The animals were randomly assigned to undergo sham operation or five-sixth nephrectomy to establish a renal hypertension model. Briefly, two-thirds of the left kidney was first removed; a week later, the right kidney was totally excised under pentobarbital anesthesia. The sham group only underwent de-capsulation of the kidneys.

Ten weeks after the final surgery, 5/6Nx rats were randomized into 7 groups (*n* = 6 per group): ➀no treatment; ➁intracerebroventricular injection (ICV) of artificial cerebrospinal fluid (aCSF) as the vehicle; ➂ICV of losartan (Sigma Chemical, 2.29 mmol/l/kg), an angiotensin II subtype 1 receptor (AT1R) antagonist; ➃ICV of farnesylthiosalicylic acid (FTS) (Cayman Chemical, 1 mmol/l/kg), a Ras inhibitor; ➄ICV of 2-(2-Amino-3-methoxyphenyl)-4H-1-benzopyran-4-one (PD98059) (Sigma Chemical, 200 μmol/l/kg), a selective MEK inhibitor that effectively inhibits ERK1/2 phosphorylation; ➅ICV of 4-(4-Fluorophenyl)-2-(4-methylsulfinylpheyl)-5-(4-pyridyl)-1H-imidazole (SB203580) (Sigma Chemical, 200 μmol/l/kg), a p38MAP kinase inhibitor; ➆ICV of N-Benzyloxycarbonyl-Asp (OMe)-Glu (OMe)-Val-Asp- (OMe)-fluoro-methylketone (Z-DEVD-FMK) (Calbiochem, 1500 μmol/l/kg), a caspase-3 inhibitor. Sham operated rats (*n* = 6) with no treatment were used as normal controls.

ICV was performed with a stereotactic frame (David Kopf Instrument Inc., USA) after anesthesia with 3% pentobarbital sodium (0.15 mL/100 g body weight). A brain-infusion cannula (Brain Infusion Kit 2; ALZET Inc., USA) coupled to an osmotic pump (Model 2002; ALZET Inc., USA) was implanted into the cerebral ventricle. The coordinates were −1.0 mm posterior and 1.5 mm lateral from the midline, and 4.5 mm ventral, with respect to the bregma. Osmotic pumps were placed subcutaneously at the back of the neck. Following surgery, the wounds were carefully closed. The implanted osmotic pumps would continuously infuse aCSF or respective drugs into the lateral cerebral ventricle at 0.5 μl/h for 14 days.

### Measurements and sample collection

Ten weeks after the final nephrectomy or sham operation, rats were weighted; 24-h urine samples were collected and urinary protein excretion was assessed by the Bradford method; blood pressure was determined with a pressure transducer (Gould) placed in the femoral artery and connected to a physiologic recorder (Gilson Medical Electronics) in anesthetized rats (Li et al., 2007). Serum creatinine levels were measured on an automatic biochemical analyzer (AU480, Beckman Coulter). Plasma norepinephrine concentrations were assessed using a competitive ELISA kit using TMB (3, 3, 5, 5-TetraMethyl benzidine solution liquid MeMbrane substrate) as a substrate and finally monitored at 450 nm. Moreover, the standard range and the sensitivity of the kit are 0.2–32 ng/ml and 1.3 pg/ml, respectively (Demeditec Diagnostics, DEE5200).

Two weeks after administration of aCSF or drugs, the above measurements were performed again. Then, all animals were anesthetized with 3% pentobarbital sodium (0.15 mL/100 g body weight) and sacrificed by cervical dislocation.

Some rats were transcardially perfused with 200 ml ice-cold normal saline followed by 400 ml 4% paraformaldehyde. Then, the brains were removed and sectioned, fixed for 6 h, and dehydrated in graded alcohol. Finally, the samples were paraffin embedded and sliced in 5 μm sections for immunochemistry. To identify the position of PVN, the brains were immediately removed and1-mm thick sections were cut using a cryostat. The PVN was defined and excised from 1-mm-sections on dry ice based on an rat brain atlas (Paxinos and Watson, 1998; Figure S1). PVNs were isolated from brains in accordance with the steps above, snap frozen in liquid nitrogen, and stored at −80°C for protein and RNA extraction.

### Immunohistochemistry and immunofluorescent tunel reaction

Immunohistochemical assessment of RAS, p-ERK1/2, and Bax levels in PVN samples was performed with the avidin–biotin-peroxidase complex method. Primary antibodies were mouse anti-AGT monoclonal antibodies (1:500, Swant, Switzerland), rabbit polyclonal antibodies raised againstangiotensin II (1:400, Peninsula laboratories, USA), AT1R (1:100, Millipore, USA), and Bax (1:500, Santa Cruz, USA), and rabbit monoclonal antibodies targeting phospho-ERK1/2 (1:100, Cell Signaling Technology, USA), respectively. Biotinylated goat anti-rabbit/mouse secondary antibodies (1:200) were used for detection. Finally, sections were incubated with avidin–biotin–peroxidase complex (VECTASTAIN ABC Kit, USA) and peroxidase substrate solution (Vector Laboratories, USA) until optimal signal intensity developed. After dewaxing and hydration with graded ethanol, photographs were acquired under a microscope and analyzed using the Image-Pro Plus software. Finally, positive cells were counted in 10 low-power field at least three slices per group. Data were presented as positive cells per 10^3^ μm^2^.

Immunoflourescent TUNEL staining was performed by following steps: The sections were rehydrated and treated with 0.1% Triton-100 for 30 min at room temperature, then applied 100 uL TUNEL reaction mix from the In Situ Cell Death Detection Kit (Roche 11684817910) in a dark humidified chamber at 37°C for 60 min prior to counterstaining tissue with 4′,6-diamidino-2-phenylindole (DAPI) nuclear dye.

### Western blotting

PVN samples were homogenized in a lysis buffer and Western blot was performed as previously described (Su Z. et al., 2014). Specifically, Ras active protein (Ras-GTP) was extracted from total tissue lysates by adding 80 ug GST-Raf1-RBD per 500 ug sample and using Active Ras Pull-Down and Detection Kit (Thermo scientific, USA) and assessed by western blot immediately or stored at −20°C.

Primary antibodies used included the following: anti-AT1-R (1:200, Millipore, USA), anti-p-ERK1/2 (1:300, Cell Signaling Technology, USA), anti-p-p38 (1:500, Cell Signaling Technology, USA), anti-p38 (1:1000, Cell Signaling Technology, USA), anti-Bax (1:500, Santa Cruz Biotechnology), anti-Bcl-2 (1:200, Santa Cruz Biotechnology), anti-Cleaved caspase-3 (1:600, Cell Signaling Technology), anti-ERK1/2 (1:1000, Cell Signaling Technology, USA), anti-Ras (1:200, Abcam Cambridge, UK), anti-β-actin (1:5000, Cell Signaling Technology), anti-GAPDH (1:1000, Cell Signaling Technology). The levels of the above proteins were determined as previously described (Su Z. et al., 2014). AT1-R protein content was normalized to GAPDH amounts. Ras, p-ERK1/2, and p-p38 levels were normalized to total Ras, ERK1/2 and p38, respectively. Bax, Bcl-2 and cleaved caspase-3 protein levels were normalized to β-actin.

### Real-time PCR

Total RNA from PVN samples was extracted with TRIzol reagent (Invitrogen) according to the manufacturer's instructions. RNA concentrations were measured on a Nanodrop ND1000 spectrophotometer (NanoDrop technologies, Wilmington, DE, USA). Reverse transcription was performed with a One-Step RT-PCR kit (TaKaRa, Kyoto, Japan) according to the manufacturer's protocol. Quantitative real-time PCR was performed in a total volume of 25 μl in duplicate on a Fast Real-Time PCR system 7500 (Applied Biosystems, CA, USA) with the SYBR Premix Ex Taq kit (TaKaRa, Kyoto, Japan). Thermal cycling conditions comprised 30 s at 95°C, followed by 95°C for 5 s, 60°C for 30 s, and 72°C for 60 s for 40 cycles. The relative expression levels of targeted mRNAs were calculated after normalization to GAPDH mRNA using the 2^−ΔΔCT^ method. The primer sets used are summarized in Table 1.

**Table 1:** **Primers used for quantitative Real-time PCR**.

	**Forward**	**Reverse**
AT1R	5′-CAGTGTGCGCGTTTCATTATG-3′	5′-TGGTAAGGCCCAGCCCTAT-3′
Bax	5′-TGGTTGCCCTTTTCTACTTTG-3′	5′-GAAGTAGGAAAGGAGGCCATC-3′
Bcl-2	5′-CTGGTGGACAACATCGCTCTG-3′	5′-GGTCTGCTGACCTCACTTGTG-3′
GAPDH	5′-TGCCAAGTATGATGACATCAAGAA-3′	5′-AGCCCAGGATGCCCTTTAGT-3′

### Statistical analysis

All analyses were performed with SPSS 19.0 for Windows. Data are mean ± standard error of the mean (SEM). Continuous variables between groups were compared by one-way analysis of variance, followed by Least Significant Difference (LSD test). The level of statistical significance was set at 0.05.

## Results

### Successful establishment of a rat model of chronic renal hypertension

Ten weeks after the 5/6 nephrectomy (5/6 Nx), body weight, 24 h urinary protein excretion, systolic blood pressure, serum creatinine, and plasma norepinephrine in model rats were evaluated, in comparison with sham-operated controls (Table S1, *p* < 0.05). Plasma norepinephrine concentrations were used as an indicator of sympathetic nerve activity (Zoccali et al., 2002). As shown in Table S1, 5/6Nx rats showed similar body weights, but significantly higher 24 h urine protein excretion as well as systolic blood pressure, serum creatinine, and plasma norepinephrine (*p* < 0.05). These findings indicated a successful establishment of a rat model of chronic renal hypertension.

### General characteristics of the hypertensive animals after 14 days of treatment with various agents

There were no differences among rats with renal hypertension administered aCSF and various agents, in body weight and serum creatinine (Table 2, *p* < 0.05). Compared with the aCSF group, intracerebroventricular injection with losartan, FTS, PD98059, and Z-DEVD-FMKICV resulted in significantly decreased urinary protein, systolic blood pressure and plasma norepinephrine levels (Table 2, *p* < 0.05). However, the p38 inhibitor SB203580 showed no effects on the above parameters.

**Table 2:** **General characteristics before and after 14 days of blockade with various approaches in renal hypertension rats or sham rats^A^**.

**Group**	**BW**	**SBP**	**Scr**	**UPE**	**Plasma NE**
Sham 5/6Nx	495.6±5.0	124.7±3.2	41.9±0.6	8.6±0.5	235.4±21.9
10 weeks	538.3±10.4	148.8±2.1^B^	106.6±7.2^B^	28.7±3.0^B^	589.5±17.0^B^
12 weeks	498.6±7.9	150.3±3.2^B^	105.7±8.2^B^	22.4±1.8^B^	655.0±37.0^B^
5/6Nx+ i.c.v. aCSF	489.7±6.8	146.0±2.1^B^	106.9±7.2^B^	22.4±1.2^B^	608.1±48.1^B^
5/6Nx+ Losartan	490.0±3.5	119±1.5^C^	100.4±2.8	11.6±0.8^C^	302.1±3.0^C^
5/6Nx+ FTS	493.3±2.9	119±2.3^C^	106.1±6.6	12.2±0.6^C^	276.5±23.2^C^
5/6Nx+ PD98059	490.7±4.5	122.0±2.1^C^	100.9±2.2	12.7±0.4^C^	240.7±15.4^C^
5/6Nx+ SB203580	496.0±5.2	140.7±0.89	104.7±9.0	21.9±1.6	573.4±30.2
5/6Nx+ Z-DEVD-FMK	501.0±7.1	128.8±2.2^C^	103.3±9.1	12.2±0.4^C^	312.5±26.1^C^

A *Data from 3 independent experiments are expressed as mean ± SD (n = 6 for each group)*.

B *p < 0.05 vs. Sham group*,

C *p < 0.05 vs. 5/6Nx + i.c.v CSF group*.

*5/6 Nx: five-sixth nephrectomy; BW, body weight; SBP, systolic blood pressure; Scr, serum creatinine; UPE, urinary protein excretion; Plasma NE, Plasma norepinephrine. Losartan (angiotensin II receptor type 1 (AT1R) antagonist), FTS (farnesyl thiosalicylic acid, Ras inhibitor), PD98059 (MEK inhibitor), SB203580 (p38 inhibitor), Z-DEVD-FMK (caspase-3 inhibitor)*.

### AT1R, Ras-GTP, p-ERK1/2, and p-p38 levels in PVN specimens from model and sham-operated rats

**Figure 2:**
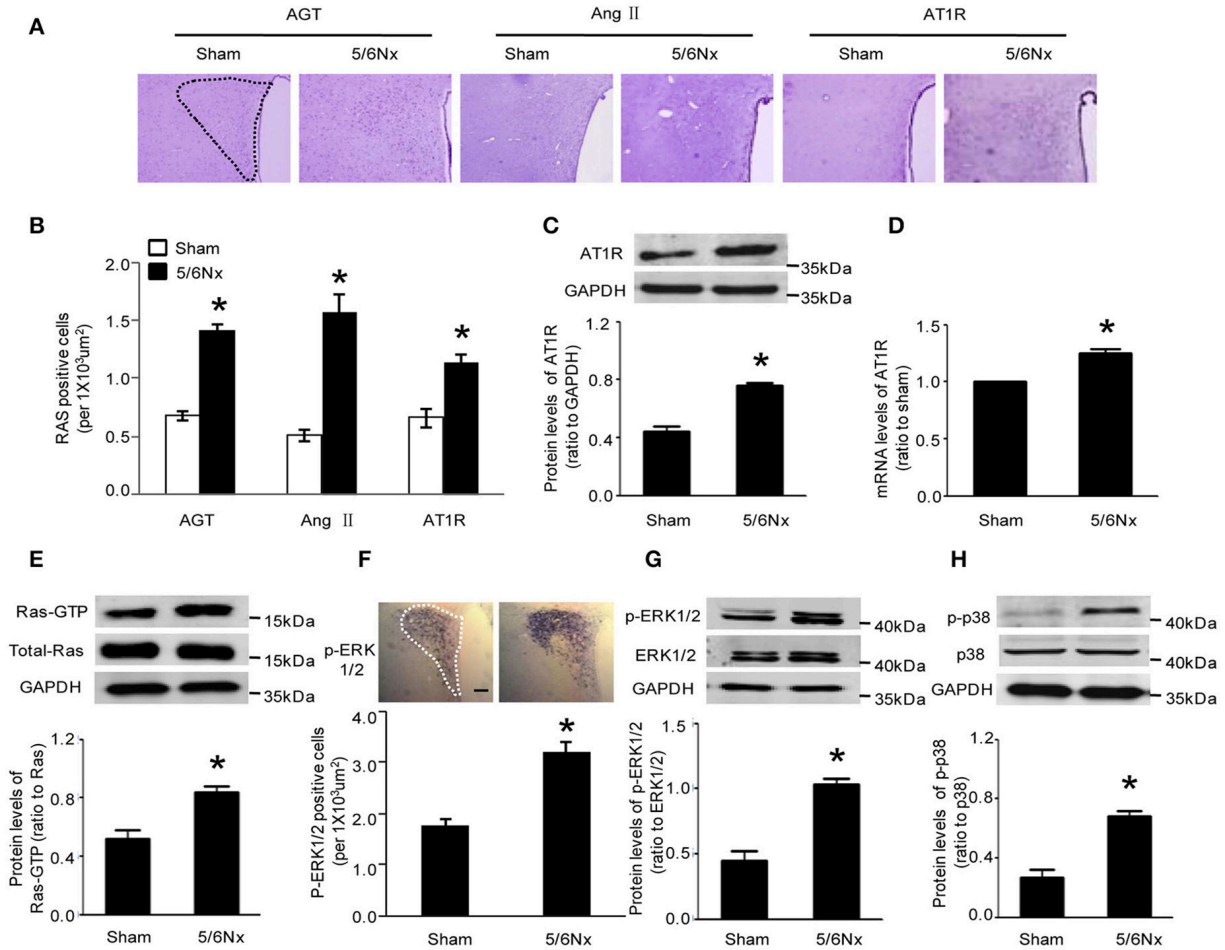
**Expression levels of AGT, AngII, AT1R, Ras-GTP, p-ERK, and p-p38 in PVN of sham-operated and 5/6Nx rats. (A)** Representative immunochemistry micrographs of samples from sham and 5/6Nx groups for AGT, AngII, and AT1R respectively (PVN nucleus in this picture was circled by dotted line). **(B)** Representative quantitative statistics of samples of sham and 5/6Nx groups for AGT, AngII, and AT1R, respectively. **(C,D)** Representative protein expression and mRNA level of AT1R were assessed in sham and 5/6Nx groups, by RT-PCR and western blot, with GADH used for normalization. **(E,F)** Ras-GTP and total Ras protein expression levels assessed by western blot and immunohisochemistry technique in sham and 5/6Nx groups, respectively. **(G,H)** p-ERK 1/2, ERK 1/2, p-P38, and P38 protein expression levels in sham and 5/6Nx groups. Data are mean ± SEM (*n* = 6 per group). ^*^p < 0.05 vs. sham group. 5/6 Nx: five-sixth nephrectomy.

Immunochemistry, western blot and real-time PCR were used to assess the expression of AGT, Ang II, and AT1R. Compared with the values obtained for sham-operated rats, AGT, Ang II, and AT1R amounts in PVN from rats with renal hypertension were significantly higher (Figures 2A–D, *p* < 0.05). Western blot and immunochemistry were used to detect Ras-GTP, p-ERK1/2, and p-p38. Compared with sham-operated rats, the model animals showed significantly higher Ras-GTP, p-ERK1/2 and p-p38 protein levels in PVN samples (Figures 2E–H, *p* < 0.05).

### Neuronal apoptosis in PVN specimens from model and sham-operated rats

**Figure 3:**
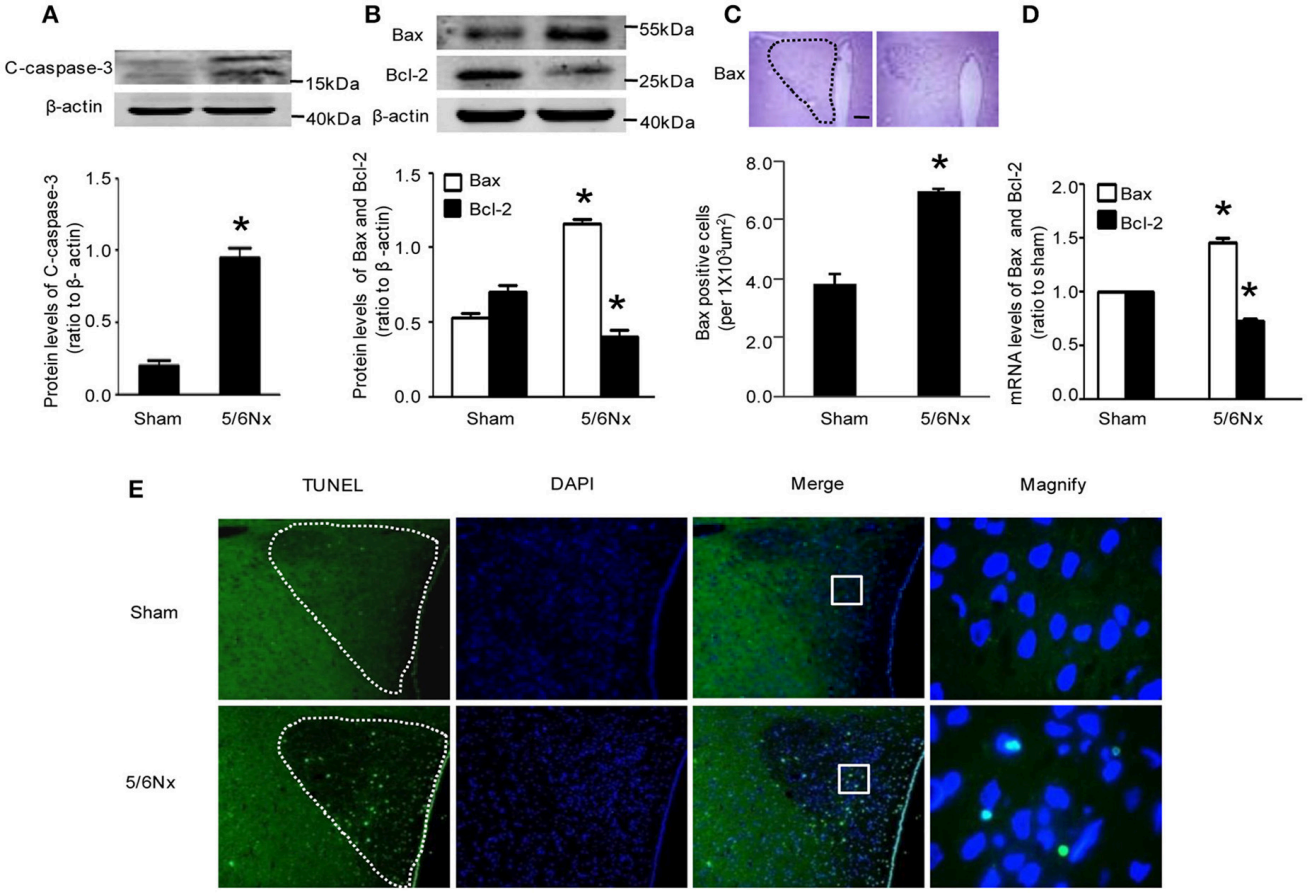
**Apoptosis markers' change in PVN samples from rats with renal hypertension and sham operation. (A)** C-caspase-3 protein levels in sham and 5/6Nx rats. **(B)** Bax and Bcl-2 protein levels in sham and 5/6Nx rats. **(C)** Representative immunochemistry micrographs and quantitative statistics of Bax in sham and 5/6Nx groups. (PVN nucleus in this picture was circled by dotted line). **(D)** Bax and bcl-2 mRNA levels in sham and 5/6Nx groups, by real-time PCR, with GADH used for normalization. **(E)** TUNEL staining in sham and 5/6Nx groups (PVN nucleus in this picture was circled by dotted line). Data are mean ± SEM (*n* = 6 per group). ^*^p < 0.05 vs. sham group. 5/6 Nx: five-sixth nephrectomy. C-caspase-3, cleaved-caspase-3.

Immunochemistry, western blot and real-time PCR showed that cleaved caspase-3 and Bax amounts in PVN from model rats were significantly higher compared with the values obtained for sham-operated rats Bcl-2 expression was reduced in the model rats, both at the gene and protein levels, although the latter did not reach statistical significance (Figures 3A–D, *p* < 0.05). TUNEL staining is a well-defined method for detecting apoptotic DNA fragmentation in tissue. Figure 3E demonstrated that TUNEL-positive cells apparently increased in the hypothalamus PVN of 5/6Nx rats compared to sham rats.

The observations after nephrectomy and subsequent drug treatments suggested that increased apoptosis of central nervous system neurons may play a crucial role in elevating blood pressure in case of kidney damage, possibly by relieving the constraints on the sympathetic nervous system; upregulated AT1R, Ras, ERK1/2, and caspase-3 signaling pathways in the CNS may constitute the underlying mechanisms at the molecular level. However, p38 MAPK activation appeared to play no decisive role in hypertension.

### Regulatory associations of RAS, Ras, and ERK with apoptosis in the PVN from rats with renal hypertension

**Figure 4:**
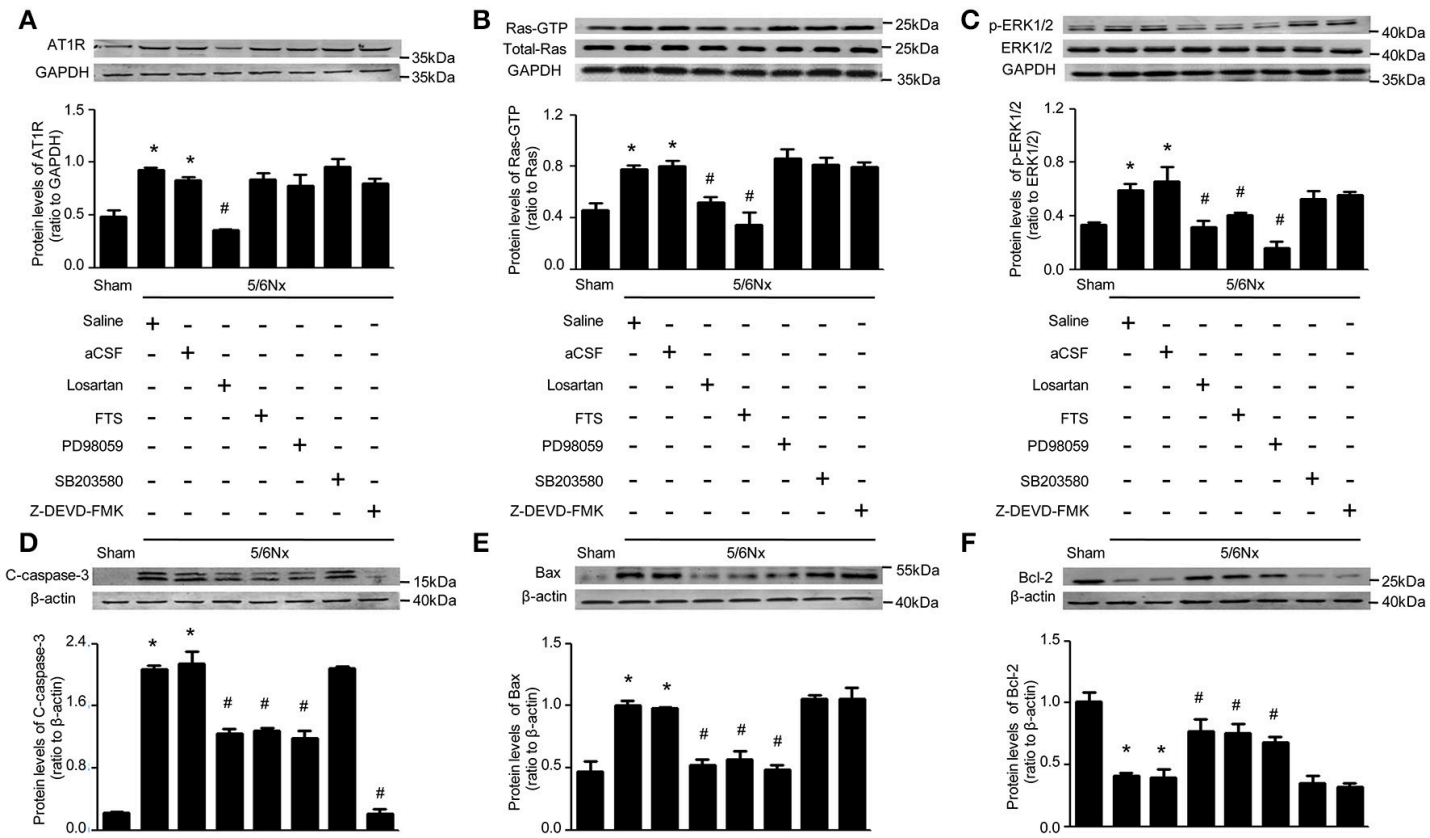
**Protein levels RAS, Ras, ERK, and apoptosis markers in PVN samples after 14 days of blockade with various approaches in renal hypertension and sham rats**. **(A)** AT1R protein levels in sham and 5/6Nx rats treated with intracerebroventricular injection (ICV) various inhibitors. **(B)** Ras-GTP and total Ras protein levels in sham and 5/6Nx rats treated with intracerebroventricular injection (ICV) various inhibitors. **(C)** p-ERK1/2 and ERK 1/2 protein levels in sham and 5/6Nx rats treated with intracerebroventricular injection (ICV) various inhibitors. **(D)** Cleaved caspase-3 protein levels in sham and 5/6Nx rats treated with intracerebroventricular injection (ICV) various inhibitors. **(E)** Bax protein levels in sham and 5/6Nx rats treated with intracerebroventricular injection (ICV) various inhibitors. **(F)** Bcl-2 protein levels in sham and 5/6Nx rats treated with intracerebroventricular injection (ICV) various inhibitors. Data are mean ± SEM (*n* = 6 per group). ^*^p < 0.05 vs. sham group; ^#^*p* < 0.05 vs. 5/6Nx+aCSF group. Losartan (Angiotensin II receptor type 1 (AT1R) antagonist), FTS (farnesyl thiosalicylic acid, Ras inhibitor), PD98059 (MEK inhibitor), SB203580 (p38 inhibitor), Z-DEVD-FMK (caspase-3 inhibitor).

To determine the possible regulation mechanism of AT1R on blood pressure and sympathetic nerve activity, AT1R protein expression in PVN of rats with renal hypertension was assessed after intracerebroventricular injection with aCSF, losartan, FTS, PD98059, and Z-DEVD-FMK, by western blot. Interestingly, high AT1R levels in PVN of model rats were decreased only by ICV losartan (*p* < 0.05). Meanwhile, FTS, PD98059, SB203580, and Z-DEVD-FMK administered by ICV showed no effects on AT1R protein expression (Figure 4A).

Meanwhile, Ras activity was significantly lower in samples from model groups treated (ICV) with losartan and FTS (*p* < 0.05); however, ICV treatment with PD98059, SB203580, and Z-DEVD-FMK yielded no effects on Ras activity (Figure 4B). These findings demonstrated that AT1R regulated Ras activity, with the remaining blocking agents failing, in the PVN of rats with renal hypertension.

Interestingly, ERK1/2 phosphorylation was significantly lower in PVN samples from model rats that received ICV injection of losartan, FTS, and PD98059 (*p* < 0.05), with SB203580 and Z-DEVD-FMK showing no effects on ERK1/2 activity (Figure 4C). These findings demonstrated that both AT1R and Ras regulated ERK1/2 phosphorylation, unlike the remaining blocking agents, in the PVN of rats with renal hypertension.

The effects of these blocking agents were evaluated on markers of cell apoptosis. As shown in Figure 4, Bax and cleaved caspase-3 levels were significantly higher in PVN samples from model rats compared with Sham-operated animals, while Bcl-2 amounts were significantly lower. Meanwhile ICV injection of losartan, FTS, and PD98059 in rats with renal hypertension significantly reduced Bax amounts, and markedly increased Bcl-2 levels. In addition, ICV injection of losartan, FTS, PD98059, and Z-DEVD-FMK in rats with renal hypertension significantly reduced cleaved caspase-3 amounts (Figures 4D–F, *p* < 0.05).

## Discussion

This study demonstrated that chronic renal disease due to nephrectomy would lead RAS activation in PVN of the hypothalamus, which further induced neuronal apoptosis through the Ras/ERK1/2 signaling pathway. The neuronal apoptosis in PVN may deregulate sympathetic activity and bring about renovascular hypertension.

Brain RAS in blood pressure regulation have attracted more and more attention for decades. It has been shown that AT1R expression increased in the cardiovascular regulatory region, with an enhanced sensitivity to Ang II in animal models with neurogenic hypertension (Xia et al., 2013). Hyperactivity of Ang II/AT1R in the CNS leads to neurogenic hypertension (Biancardi et al., 2014). In the present study, excessive activation of RAS was found in the PVN from renal hypertensive rats, as reflected by increased expression levels of AGT, Ang II, and AT1R. And blocking the brain RAS with AT1R antagonist losartan could provide relief from high systolic blood pressure. These findings were in accordance with our previous work concerning the relation of RAS activation in PVN and chronic renal failure. We has also demonstrated that increased central oxidative stress leads to an increase in sympathetic activity, and blockade of oxidative stress with intracerebroventricular administration of antioxidant tempol reduced sympathetic activity in rats with chronic renal failure fed with high salt diet (Cao et al., 2015). In addition, Braga et al. reported that chronic peripheral Ang-II infusion in mice produces a slow developing hypertension, which is accompanied by superoxide accumulation in the RVLM and increased sympathetic activity (Braga et al., 2011). Furthermore, norepinephrine, which is a biomarker of sympathetic activation, stimulated apoptosis in adult rat ventricular myocytes *in vitro* (Communal et al., 1998). Thus, Ang-ROS pathway may increase sympathetic activity which finally result in apoptosis.

*In vitro* experiments had suggested that Ang II activation is responsible for apoptosis and proliferation of endothelial and epithelial cells in a model of malignant hypertension (Efrati et al., 2007). Ang II was also found to play a critical role in the development of renal cell apoptosis in Ang II-infused hypertension, while losartan decreases apoptosis (Aizawa et al., 2001). In addition, our study explored the relationship of RAS activation and elevated apoptosis in the context of nervous system cells. We discovered that hypertensive rats showed pro-apoptotic mechanism activation in PVN, as reflected by increased levels of Bax and caspase-3, decreased amount of Bcl-2, and elevated TUNEL-positive cells, which suggested neuronal loss in PVN after 5/6Nx operation. And the fact that intracerebral injection of Z-DEVD-FMK could reduce blood pressure and lowered plasma norepinephrine level had clearly indicated the PVN apoptosis induced by AT1R activation made important contributions to pathogenesis of renal hypertension, possibly through increasing sympathetic activity.

This study also showed that Ras, p-ERK1/2 (p44/42 MAPK) and p38 MAPK signaling were all activated in the PVN of rats with renal hypertension. The MEK/ERK1/2 signaling plays an important role in the regulation of the renin-angiotensin system toward the sympathetic nervous system, in the brain of heart failure rats (Wei et al., 2008b). This role of ERK1/2 in Ang II-induced hypertension was further demonstrated by the observation that injection of the MEK inhibitor PD98059 bilaterally into the RVLM dramatically lowers Ang II-infused hypertension (Seyedabadi et al., 2001). In other words, hypertension resulted from RAS activation in the ventrolateral medulla is dependent on ERK1/2 phosphorylation. Our research work discovered that for the central nervous regulation of sympathetic activity and blood pressure in PVN, p-ERK1/2 was also essential in bridging RAS activation and hypertension development; while the increase of p-p38 appeared only the concomitant consequence but not the decisive factor. Another study had showed that MAPK pathway in turn plays an important role in up-regulation of AT1R (Wei et al., 2008a). Yet, we found in the renal hypertensive rat model, the positive regulation of AT1R toward p-ERK1/2 was unidirectional.

Ras proteins are expressed in almost all cell types, including fibroblasts and muscle cells, and activate signal transduction pathways that regulate cellular proliferation, differentiation, and survival (Schwartz et al., 2007). In a study assessing non-small cell lung cancer (Pelaia et al., 2012), Ras inhibitors were found to inhibit ERK1/2 activation and apoptosis. What's more, previous work had illustrated that the MAPK pathway induced by Ras was involved in apoptosis regulation of RVLM, which resulted in sympathetic overactivity and hypertension development upon AT1R activation (Kishi et al., 2010). The present study revealed similar associations of AT1R, Ras, and ERK1/2 in apoptosis regulation of the PVN as in the RVLM, while with regard to the pathogenesis of renal hypertension rather than stroke-prone spontaneous hypertension.

The present study has some limitations. After 2-week infusion of drugs in PVN, the SNA and blood pressure was decreased by ICV infusion of the AT1R antagonist, Ras inhibitor, MEK inhibitor, and caspase-3 inhibitor, but we did not assess the changes in RVLM, which may also be important to the regulation of renal hypertension. Therefore, we cannot exclude the effects of these drugs in other brain sites, and the role of other autonomic nuclei in neural control of SNA and blood pressure needs further study. In addition, we did not measure the concentrations of ICV infused drugs in the blood, and thus cannot exclude some spillover effects in the periphery. However, the ICV dosage of losartan were >500-fold lower than the dose commonly used in the periphery by gastrogavage and were chosen from preliminary studies in which intravenous injection of these doses had no detectable effects on AngII-induced increase in blood pressure or circulating norepinephrine levels (Cao et al., 2015).

Taken together, our findings suggested that RAS system mainly composed of angiotensinogen, Ang II, AT1R, was activated in the PVN of renal hypertensive rats; those changes would mediate neuronal apoptosis through the Ras/ERK1/2 pathways, regulating sympathetic activity and blood pressure, which played a crucial role in the pathogenesis of renal hypertension. Inhibition by intracerebroventricular infusion of ARB in the PVN may be a novel therapeutic approach to sympathoexcitation in hypertension. Moreover, angiotensin II and ROS are important modulating factors regulating SNA, which is involved in hypertension and heart failure. We consider that neural apoptosis in the PVN is a novel target for the treatment of cardiovascular diseases exhibiting increased SNA and blood pressure.

## Author contributions

AL, HZ, MZ, LT conceived and designed the research; YL, JL, and MQ performed the experiments; LL and MZ analyzed the data. AL, ML, HZ, LT wrote and revised the manuscript.

### Conflict of interest statement

The authors declare that the research was conducted in the absence of any commercial or financial relationships that could be construed as a potential conflict of interest.
