# Supplementary material for: Increased Apoptosis in the Paraventricular Nucleus Mediated by AT1R/Ras/ERK1/2 Signaling Results in Sympathetic Hyperactivity and Renovascular Hypertension in Rats after Kidney Injury
Source: Front Physiol. 2017 Feb 2;8:41. doi: 10.3389/fphys.2017.00041 (PMC5288364; doi:10.3389/fphys.2017.00041)
Supplement: Supplementary file 1 [file Presentation1.pdf]

Increased apoptosis in the paraventricular nucleus mediated by AT1R/Ras/ERK1/2 signaling results in sympathetic hyperactivity and renovascular hypertension in rats after kidney injury

Hongguo Zhu[#], Lishan Tan[#], Yumin Li, Jiawen Li, Minzi Qiu, Lanying Li, Mengbi Zhang, Min Liang*, Aiqing Li*

Department of Nephrology, Nanfang Hospital, Southern Medical University, State Key Laboratory of Organ Failure Research, National Clinical Research Center of Kidney Disease, Guangzhou, 510515, Guangdong Province, China

[#]Hongguo Zhu and Lishan Tan contributed equally to this work.

***Correspondence**: Aiqing Li, Phone: 86-20-62787973; FAX: 86-20-87281713; E-mail: liaiqing@smu.edu.cn; Min Liang, Phone: 86-20-62787973; FAX: 86-20-62787973; E-mail: nfyylm@163.com

This file includes:

Table S1 & Figure S1

Table S1. General characteristics of rats 10 weeks post 5/6 nephrectomy or sham operation [A]

	Sham	5/6 Nx
Body weight (g)	559.3±8.8	538.3±10.4
SBP (mmHg)	126.3±2.5	148.8±2.1[B]
Scr (μmol/l)	32.3±5.4	106.6±7.2[B]
UPE (mg/24h)	13.7±2.8	28.7±3.0 [B]
Plasma NE (pg/ml)	239.5±9.4	589.5±17.0[B]

[A] Data from 3 independent experiments are expressed as mean ± SD (n = 6 in each group); [B] $p < 0.05$ *vs*. sham group; 5/6 Nx: five-sixth nephrectomy; SBP, systolic blood pressure; Scr, serum creatinine; UPE, urinary protein excretion; Plasma NE, Plasma norepinephrine.

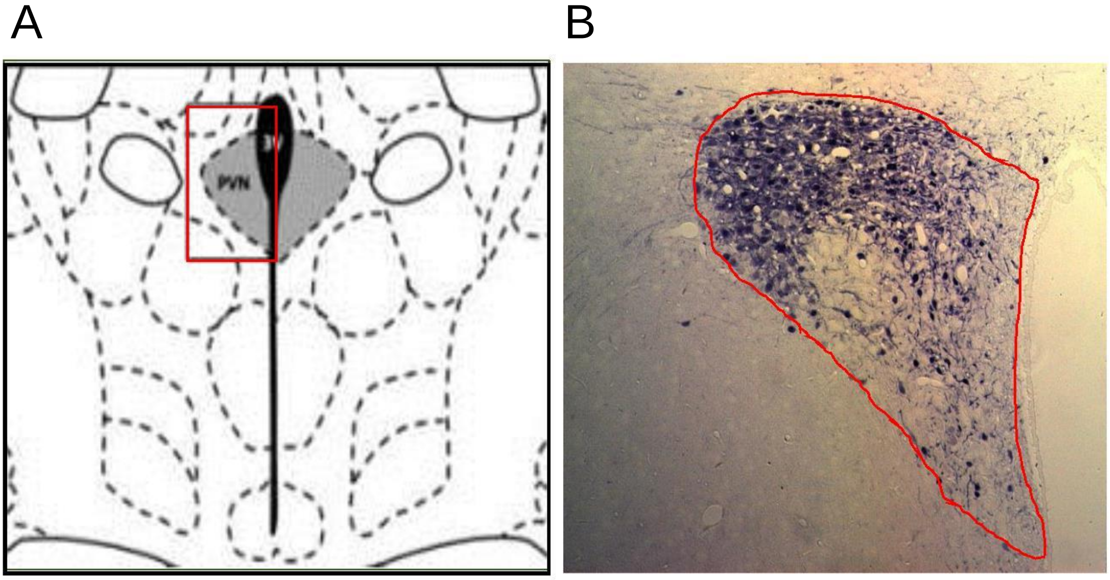

Figure S1. PVN structure was shown in the schematic. (A) The location of PVN in the brain atlas; (B) Immunochemistry of p-ERK1/2 in the PVN (area within the red line is the area of interest).
